# Effects of medical and surgical treatment on vitamin D levels in obesity

**DOI:** 10.1371/journal.pone.0292780

**Published:** 2023-12-22

**Authors:** Ala Mejaddam, Gudrún Höskuldsdóttir, Frida Lenér, Ville Wallenius, Penelope Trimpou, Lars Fändriks, Karin Mossberg, Björn Eliasson, Kerstin Landin-Wilhelmsen

**Affiliations:** 1 Department of Internal Medicine, Sahlgrenska University Hospital/Östra, Gothenburg, Sweden; 2 Department of Internal Medicine and Clinical Nutrition, Institute of Medicine, Sahlgrenska Academy, University of Gothenburg, Gothenburg, Sweden; 3 Section for Endocrinology and Diabetology, Department of Medicine, Sahlgrenska University Hospital, Gothenburg, Sweden; 4 Department of Public Health and Community Medicine Primary Health Care, Institute of Medicine, Sahlgrenska Academy, University of Gothenburg, Gothenburg, Sweden; 5 Department of Surgery, Institution for Clinical Sciences, Sahlgrenska Academy, University of Gothenburg, Gothenburg, Sweden; National Healthcare Group, SINGAPORE

## Abstract

**Introduction:**

Persons living with obesity treated with bariatric surgery are at a high risk of developing nutritional deficiencies. The primary aim of this observational cohort study was to compare vitamin D levels in patients two years after bariatric surgery (Roux-en-Y gastric bypass/RYGB and sleeve gastrectomy/SG) with a very low-energy diet (VLED). The same subjects were also compared with a population sample from the same region at baseline. The primary hypothesis was that surgery, especially RYGB, would lead to an increased prevalence of vitamin D deficiency compared to subjects treated with VLED. 971 individuals eligible for surgical, RYGB (n = 388), SG (n = 201), and medical treatment (n = 382), in routine care, were included consecutively between 2015 and 2017. A random population sample from the WHO-MONICA project was used as a reference, (n = 414). S-calcium, S-25(OH)D (vitamin D), and S-PTH (parathyroid hormone) were measured in all persons with obesity at baseline and two years after treatment (n = 713). Self-reported use of vitamin D and calcium supplementation was registered.

**Results:**

Vitamin D deficiency (S-25(OH)D <25mmol/l) was found in 5.2% of the persons with obesity at baseline versus 1.7% of the general population (SMD>0.1). S-25(OH)D increased for all treatment groups but was higher in RYGB and SG (SMD>0.1, standardized mean difference). Thirteen subjects (1.8%) had vitamin D deficiency after obesity treatment.

**Conclusion:**

Surgical intervention for obesity followed by vitamin D supplementation was not associated with a higher risk for vitamin D deficiency, irrespective of surgery type, compared to individuals on medical treatment. However, persons living with obesity seeking weight loss treatment are more likely to have deficient vitamin D levels compared to the general population.

## Introduction

Obesity is a major health issue globally and together with obesity-related diseases it imposes a great challenge for the public health [1]. Treatment options for obesity include bariatric surgery or a non-surgical approach with pharmacotherapy, and behavioral and dietary changes. There is comprehensive evidence today that bariatric surgery is the most effective method to ensure long-term weight loss, and reduce overall mortality and co-morbidities [2, 3]. Roux-en-Y gastric bypass (RYGB) and sleeve gastrectomy (SG) are the most common surgical methods [3, 4], and while both achieve comparable outcomes regarding weight loss and beneficial effects on comorbidities [2, 5, 6], they are also accompanied by different complications [7]. The altered anatomy and physiology of the gastrointestinal tract with bariatric surgery leads, among other things, to a lifetime susceptibility to developing nutritional deficiencies such as calcium and vitamin D malabsorption [7–9].

There is a substantial body of evidence on the risk of vitamin D deficiency and secondary hyperparathyroidism as a consequence of bariatric surgery [10–12]. Data on vitamin D has mainly been reported for RYGB, which until recently was the most common procedure for weight loss. The risk for malabsorption, and vitamin D deficiency, is considered higher after RYGB because of the bypass of the duodenum and proximal jejunum [13]. In a comprehensive literature review by Peterson et al., preoperative deficiency ranged from 13% to 90% and remained similar after surgery, and was highest after RYGB [14]. However, studies comparing RYGB and SG are few with some showing higher rates of vitamin D deficiency after RYGB [10, 15], and others showing no difference between RYGB and SG [16].

Individuals with obesity are not all interested in, nor considered eligible for surgical treatment, and instead are offered medical treatment with well-structured diets. Whether individuals undergoing medical treatment are also at a higher risk for developing vitamin D deficiency and secondary hyperparathyroidism is not known.

The primary hypothesis was that surgery, especially RYGB would lead to an increased prevalence of vitamin D deficiency compared to individuals treated with a very low-energy diet (VLED). Secondly, we hypothesized that individuals with obesity seeking treatment are more likely to suffer from vitamin D deficiency than the general population.

This study is a sub-study of the BAriatric surgery SUbstitution and Nutrition study (BASUN) [17], which is an ongoing 10-year non-randomized prospective intervention trial, with an overall aim to address the differences in effects and complications between surgical and medical treatment.

## Method

### Study setting

This is an observational cohort study with a population-based sample as a reference. It is based on two studies; i) an ongoing 10-year prospective cohort study including individuals with obesity called the BAriatric surgery SUbstitution and Nutrition Study (BASUN) [17] which is compared with ii) a population-based cohort study conducted by the World Health Organization (WHO) called the MONItoring of trends and determinants in CArdiovascular disease (WHO MONICA) [18, 19]. All subjects completed their medical examinations and questionnaires at the Sahlgrenska University Hospital, Gothenburg, Sweden. The BASUN trial is registered in Clinical trials (March 03, 2015; NCT03152617).

### Subjects

The design of the BASUN study has recently been described in detail [17]. In summary, subjects with a body mass index (BMI) ≥40 kg/m^2^ or BMI ≥ 35 kg/m^2^ with obesity-related comorbidity were consecutively recruited to BASUN from the Regional Obesity Center (ROC) at the Sahlgrenska University Hospital in Region Västra Götaland, a county in western Sweden [17]. Recruitment started on 2015-05-01. The subjects were then appointed to surgical or medical treatment based on individual preferences and eligibility according to the clinical guidelines for bariatric treatment [20]. Surgical intervention was either RYGB or SG. Medical treatment consisted of a very low-energy diet for 12 up to 20 weeks depending on BMI at baseline. This was followed by a period of food reintroduction but with continued caloric restriction for up to 12 months. The first follow-up was conducted two years after treatment. At the start of the study, neither GLP-1 analogs nor naltrexone/bupropion were reimbursed for the treatment of obesity by the state which is why subjects with medical treatment only received VLED [21].

In total, 1122 individuals met the inclusion criteria, consented to participation, and underwent examination at baseline. Individuals who were eligible for treatment but lacked adequate knowledge of the Swedish language were excluded. 85% of the population in BASUN were born in Sweden.

### Controls

As part of the third population screening by the World Health Organization MONitoring of Trends and Determinants in CArdiovascular disease (WHO MONICA) in 1995, a random population sample of men and women (participants 1616, aged 25–64 years) was recruited from the Gothenburg city census, Sweden [18, 19]. From the 1995 cohort, a subset of individuals was randomly selected and invited for a re-examination in 2008, and 414 subjects participated in the re-evaluation of hormone and bone measurements, a 63% participation rate [18]. The re-examination started on 2007-10-08. The non-attendees were those who were deceased, did not reply or could not be traced, declined consent, or failed to appear in the clinic [18]. This population sample has previously been used as a reference population for the BASUN cohort [22, 23].

### Social variables and anthropometric data

Data regarding height, body weight, and BMI at baseline was collected similarly in both groups [17, 18]. Height was measured without shoes to the nearest 1 cm and body weight was measured to the nearest 0.1 kg. BMI was calculated as body weight divided by height squared. According to current definitions, BMI >25kg/m^2^ is considered overweight, BMI >30 kg/m^2^ is considered obese, and BMI ≥40 kg/m^2^ is severe obesity [24]. Data on age, sex, education level, marital status, and smoking habits were recorded through interviews in both study groups. The degree of physical activity was measured during leisure time and is based on a 4-point ordinal scale from the Saltin Grimby questionnaire in both study groups. The lowest grade of 1 indicates complete inactivity and the highest grade of 4 indicates strenuous activity several times a week [25].

### Biochemical analyses

All venous blood samples were collected in the morning after an overnight fast. Serum levels of 25-hydroxyvitamin D (S-25(OH)D; nmol/l) and intact parathyroid hormone (S-PTH; pmol/l) were measured at the accredited laboratory for clinical chemistry at the Sahlgrenska University Hospital in both study groups. Serum levels of ionized calcium (mmol/l) were only measured in the BASUN study group. For the BASUN and WHO-MONICA study, S-25(OH)D was measured with a radioimmunoassay 125I RIA kit (DiaSorin, Stillwater, MN, USA). The coefficient of variation (CV) was 6.2%. Vitamin D deficiency was defined as S-25(OH)D < 25 nmol/l and vitamin D insufficiency as S-25(OH)D ≥25< 50 nmol/l, respectively, according to the Institute of Medicine (IOM) [26, 27]. S-PTH was determined by immunoradiometric assay (Roche Cobas, Rotkreutz, Switzerland). The CV was 2.7% and the reference range was 1.6–6.9 pmol/l for S-PTH. In the WHO-MONICA study, no blood samples were collected during the summer (June–August). In the BASUN study, 21% of the participants were tested during the summer at baseline and 13% at the two-year follow-up.

### Pharmacological treatment and vitamin supplements

Self-reported vitamin D and calcium supplement use was recorded in the BASUN cohort and the population-based controls. All subjects undergoing surgical obesity treatment were recommended 400–800 IU cholecalciferol and 500 mg calcium carbonate twice daily according to ROC and national guidelines. Subjects receiving medical treatment for weight loss did not receive any specific recommendations regarding vitamin supplements. Some of these individuals reported multivitamin supplement intake containing not more than 200 IU of cholecalciferol and 120 mg of calcium citrate. These individuals were also included in the analyses as taking supplements. Self-reported use of prescription drugs was recorded and coded according to the Anatomical Therapeutic Chemical (ATC) classification system for all participants.

### Ethical considerations

The BASUN and WHO MONICA trials were both conducted according to the Declaration of Helsinki and by local statutory requirements. Both trials were approved by the Regional Ethical Review Authority in Gothenburg (Regionala Etikprövningsnämnden i Göteborg); D-number 637–14 was approved in 2014 for BASUN and the WHO MONICA project D-number 088–06 was approved in 2006, and D-number T282-11 was approved in 2011. It was also approved by the National Data Inspection Board in Sweden. All participants gave their written informed consent to participate in the studies and for publications. Only the principal investigators of the studies have access to information to identify the participants.

### Statistical methods

A power analysis was made at the start of the BASUN study. It was based on the criteria of allowing a 20% dropout and more than 80% power. Of all the parameters included in the study, ionized calcium was defined as the parameter that would require the highest number of participants to detect a significant and clinically relevant change. Therefore, this parameter was used for the power calculation, even though it is not one of the main variables in the study. Based on this, a sample size of 1400 individuals was determined to achieve 80% power [21].

Conventional methods were used to calculate means and standard deviation (SD) values for continuous variables. Categorical variables are recorded as numbers with their percentages calculated. Standardized mean difference (SMD) was used to measure the difference in group characteristics within the BASUN study and with the reference population (WHO MONICA). SMD is an expression for effect size and distance between groups and can be used for both continuous and categorical variables [28]. The changes in clinical variables at the two-year follow-up were analyzed using a linear regression model and were reported as estimated means with 95% confidence intervals. The model was adjusted for age, sex, and baseline values for each variable as they were considered to be potential confounders. A separate analysis of variance (ANOVA) with Tukey’s post hoc test was also conducted for between-treatment group analysis in the study group.

We examined the predictive value of each clinical variable on vitamin D deficiency outcome in BASUN using a machine learning algorithm with a random forest model. For each binary classification model, three thousand trees were used. A conditional permutation scheme in the random forest model was also performed to minimize the bias of correlating variables which will reflect the true impact of each of these predictor variables (party package in R) [29]. The assessed variables were then divided manually into 10 clinical domains (Anthropometry; Sex/Age; Social status; Treatment; Comorbidity; Lifestyle/habits; Biomarkers, vitamins/minerals; Biomarkers, other; Vitamin supplements; Biomarker, metabolic) and the importance of each domain was examined separately.

To follow up the results of the random forest analysis, multivariate regression was used to analyze the association between independent variables and serum levels of S-25(OH)D as well as vitamin D insufficiency at follow-up. In both models, age, treatment category, S-25(OH)D at baseline, S-PTH, and vitamin D supplement at two years were included as independent variables. Country of birth was also included in the linear regression model separately.

Before performing the random forest and multivariate regression analyses, we imputed missing observations by using multiple imputations with a chained equation (MICE) algorithm (mice package in R). At the two-year follow-up vitamin D concentrations were missing for 27% of the subjects and vitamin D and calcium supplements were missing for almost 34% of the subjects (S1 Table).

All statistical analyses were calculated using R (R Foundation for Statistical Computing, version 4.0.3) and Microsoft Excel.

## Results

**Fig 1:**
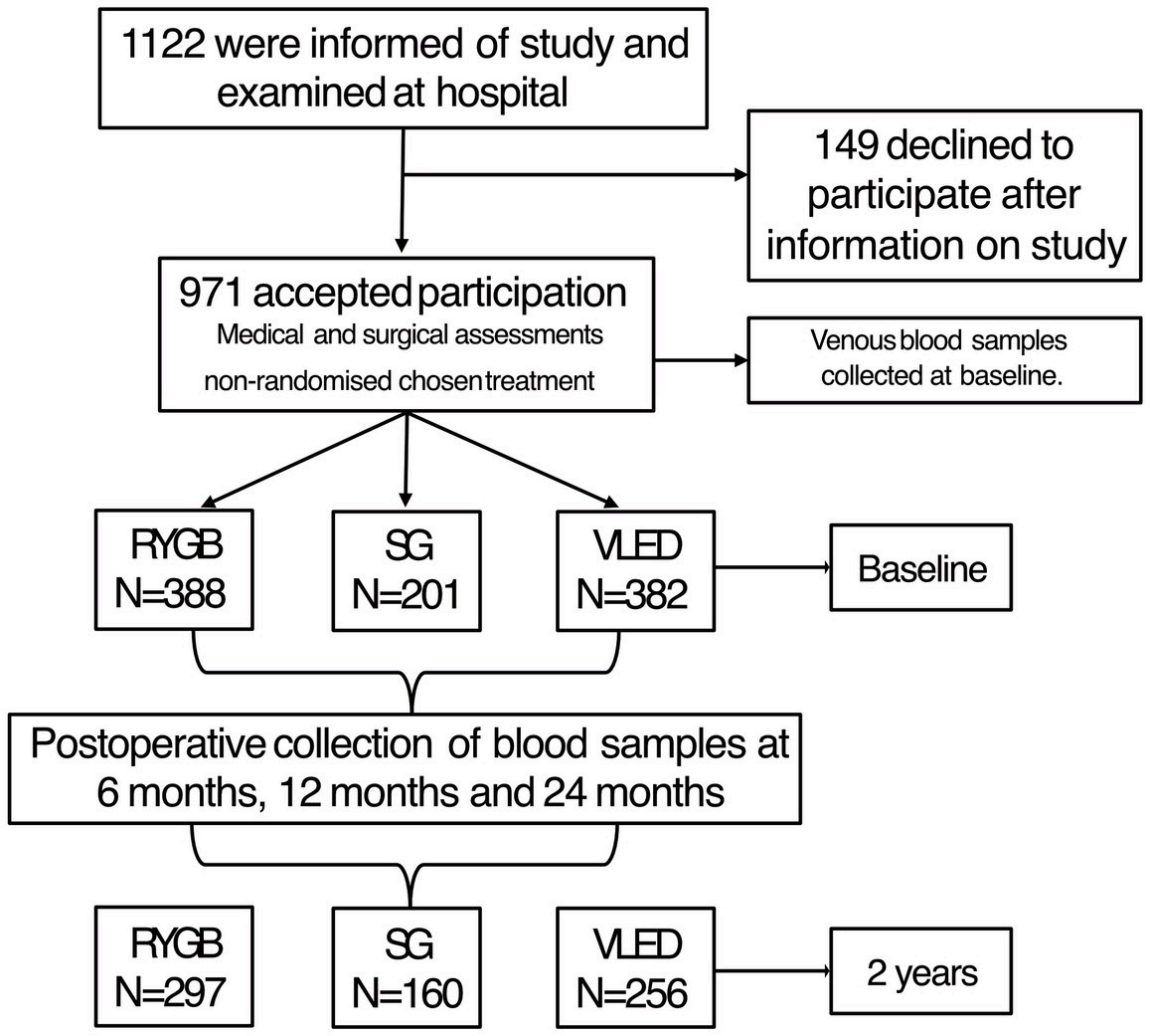
Flow chart of the BASUN study from baseline to the postoperative 2-year follow-up. RYGB; Roux-en-Y gastric bypass. SG; Sleeve gastrectomy. VLED; very low-energy diet.

In total, 971 subjects with a mean age of 43.9±12.8 years and BMI of 42.0±4.9 kg/m^2^ received treatment after inclusion in the BASUN study (Fig 1). 149 subjects chose not to continue with treatment after inclusion and they were not included in the study. A clinical description of the study population at baseline and the reference population is presented in Table 1. There were no differences between the groups regarding the distribution of sex. People in the reference population were older and had lower BMI (SMD>0.1, Table 1). In the three treatment groups of BASUN, BMI levels were comparable but the subjects in the medically treated group were older than those who underwent surgery. Individuals with obesity reported higher rates of sedentary lifestyles during leisure time compared to the reference population. The rate of cohabitation, level of education, smoking, and the number of medications are also presented in Table 1.

**Table 1:** Descriptive statistics on individuals with obesity (BASUN study) compared to a random population sample (WHO MONICA study, Gothenburg, Sweden). Data are given for all three treatment groups within BASUN (left in the column) and for the whole BASUN population (right in the column).

	RYGB (n = 388)	SG (n = 201)	VLED (n = 382)	WHO MONICA (n = 414)	SMD	BASUN (n = 971)	SMD*
**Sex, female, n (%)**	302 (78.0)	151 (75.1)	276 (72.6)	318 (76.8)	0.07	729 (75.3)	0.04
**Age, years, mean (SD)**	42.1 (11.3)	40.9 (11.0)	47.6 (14.2)	62.8 (9.4)	1.08	43.9 (12.8)	1.68
BMI, kg/m^2^, mean (SD)	42.6 (4.1)	42.8 (4.9)	41.0 (5.3)	26.8 (4.7)	1.74	42.0 (4.9)	3.19
**Married/Cohabiting, n (%)**	252 (71.6)	134 (72.0)	217 (65.2)	348 (86.4)	0.26	603 (69.2)	0.42
**Completed Secondary school, n (%)**	245 (71.6)	139 (76.4)	241 (73.0)	197 (51.8)	0.27	625 (73.2)	0.45
**Smokers, n (%)**	23 (7.1)	14 (7.8)	25 (8.9)	46 (11.3)	0.08	62 (7.9)	0.12
**Physical exercise, n (%)**					0.45		0.81
**Sedentary**	154 (43.4)	74 (40.4)	163 (47.0)	55 (13.5)		391 (44.2)	
**Moderate**	178 (50.1)	95 (51.9)	157 (45.2)	246 (60.4)		430 (48.6)	
**Regular/Heavy**	23 (6.5)	14 (7.7)	27 (7.8)	106 (26.0)		64 (7.2)	
**Number of medications, mean/person (SD)**	2.3 (2.7)	2.7 (2.9)	3.0 (3.4)	2.1 (2.3)	0.18	2.6 (3.0)	0.22
**Vitamin D supplement, n (%)**	22 (6.7)	23 (12.0)	38 (11.7)	36 (8.7)	0.11	83 (9.8)	0.04
**S-25(OH)D nmol/l, mean (SD)**	52.9 (19.6)	53.9 (20.2)	53.6 (21.3)	63.6 (25.7)	0.24	53.4 (20.4)	0.44
**S-25(OH)D (nmol/l) <25, n (%)**	22 (5.7)	12 (6.0)	16 (4.2)	7 (1.7)	0.13	50 (5.2)	0.19
**S-25(OH)D (nmol/l) <50, n (%)**	171 (44.3)	90 (45.0)	179 (47.6)	133 (32.3)	0.16	440 (45.7)	0.28
**S-PTH (pmol/l), mean (SD)**	4.7 (2.0)	5.0 (2.5)	5.2 (2.9)	5.1 (2.0)	0.11	4.9 (2.5)	0.05
**S-PTH (pmol/l) > 6.9, n (%)**	42 (11.1)	31 (15.7)	63 (17.0)	58 (14.0)	0.09	136 (14.3)	0.01

Abbreviations: WHO MONICA, World Health Organisation MONitoring of Trends and Determinants in CArdiovascular disease; RYGB, Roux-en-Y gastric bypass; SG, Sleeve gastrectomy; VLED, Very low energy diet; BMI, Body Mass Index; PTH, Parathyroid hormone; BASUN, BAriatric surgery Substitution, and Nutrition study; SD, Standard deviation; SMD, standardized mean difference. Data is given in means (SD) or n (%). SMD>0.1 is considered statistically significant.

*SMD for BASUN (n = 971) vs. WHO-MONICA (n = 414).

Less than 10% of all the individuals with obesity reported use of vitamin D supplements before treatment. This was not significantly different from the reference population (SMD<0.1). The prevalence of vitamin D deficiency (defined as S-25(OH)D <25 nmol/L) and vitamin D insufficiency (defined as S-25(OH)D <50 nmol/L) were, however, significantly lower in the reference population (SMD>0.1, Table 1). The mean level of S-25(OH)D was also significantly higher in the reference group (SMD>0.1, Table 1). Mean levels of S-PTH were less than 6.9 pmol/L in both study groups, and there was no significant difference in the rates of secondary hyperparathyroidism (defined as S-PTH >6.9 pmol/L) (SMD<0.1, Table 1).

At the two-year follow-up, 713 subjects (73.4% of those who entered the study), with a mean age of 46.8±12.3 years and mean BMI of 31.7±6.0 kg/m^2^ completed blood testing for vitamin D. Of these, 297 subjects had undergone RYGB, 160 subjects SG and 256 subjects received medical treatment with VLED (Table 2).

**Table 2:** Anthropometric data, supplementation, S-25(OH)D and S-PTH levels 2 years after gastric bypass, sleeve gastrectomy, and medical treatment.

	Gastric Bypass (n = 297)	Sleeve Gastrectomy (n = 160)	Medical treatment (n = 256)	p-value*	SMD
**Gender (female), n (%)**	232 (78.1)	118 (73.8)	183 (71.8)	0.22	0.10
**Age (years), mean (SD)**	45.1 (10.9)	43.9 (10.8)	50.5 (13.7)*	<0.001	0.36
**BMI (kg/m^2^), mean (SD)**	28.4 (4.1)	30.5 (4.7)	36.6 (5.7)	<0.001	1.09
**Number of medications, mean (SD)**	1.6 (2.3)	1.9 (2.5)	2.7 (3.1) *	<0.001	0.27
**Vitamin D supplement, n (%)**	196 (81)	101 (71)	32 (16)	<0.001	1.09
**Calcium supplement, n (%)**	222 (91)	113 (79)	12 (6)	<0.001	1.95
**S-25(OH)D (nmol/l), mean (SD)**	71.7 (21.2)	73.6 (24.2)	59.8 (22.8) *	<0.001	0.40
**S-25(OH)D (nmol/l) <25, n (%)**	5 (1.7)	1 (0.6)	7 (2.7)	0.29	0.11
**S-25(OH)D (nmol/l) <50, n (%)**	41 (13.8)	28 (17.5)	95 (37.1) *	<0.001	0.37
**S-PTH (pmol/l), mean (SD)**	4.3 (1.6)	3.8 (1.6)	4.8 (2.4)	<0.001	0.36
**S-PTH (pmol/l) > 6.9, n (%)**	20 (6.7)	9 (5.6)	35 (13.7)	0.004	0.18
**S-ionized calcium (mmol/l), mean (SD)**	1.2 (0.0)	1.2 (0.0)	1.2 (0.0)	0.69	0.05

Abbreviations: BMI, Body Mass Index; PTH, Parathyroid hormone; BASUN, BAriatric surgery Substitution, and Nutrition study. SD, Standard deviation; SMD, standardized mean difference: n.s, not significant.

Data is given in means (SD) or n (%). SMD>0.1 is considered statistically significant.

* ANOVA test for difference between treatment groups. Significant difference only between the medical treatment group and the surgery groups.

**Fig 2:**
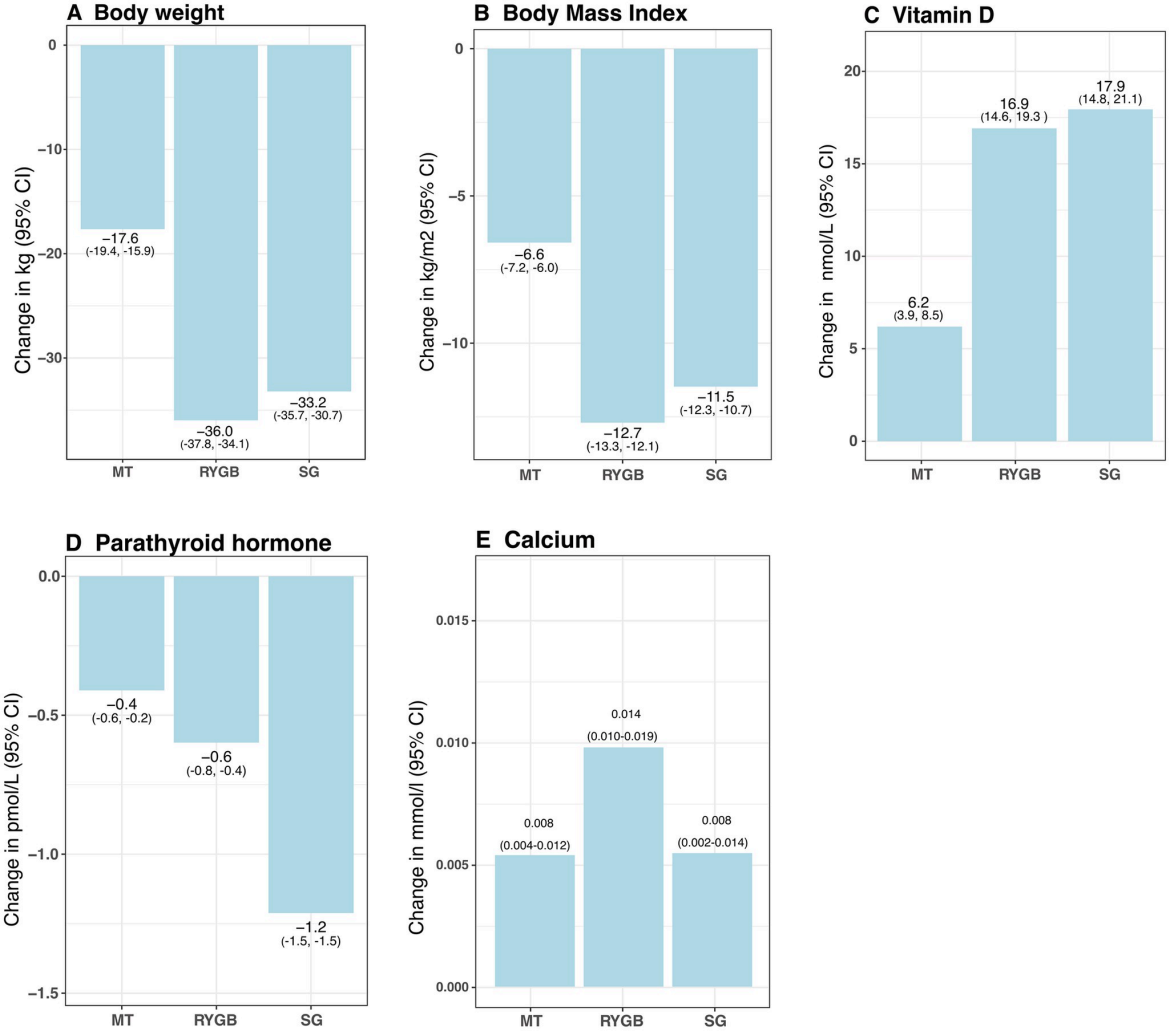
Changes in weight, body mass index (BMI), and serum levels of Vitamin D, parathyroid hormone (PTH), and calcium from baseline (before treatment) up to two years after treatment. Data is presented as estimated means with 95% CIs.

Weight changes, BMI, S-ionized calcium, S-25(OH)D, and S-PTH are shown in Fig 2. There was a significant difference in weight loss between the groups with subjects after RYGB and SG achieving the greatest weight loss.

Levels of S-25(OH)D increased from baseline for all three treatment groups with significantly higher levels in the surgically treated subjects compared to subjects on VLED (Fig 2). The corresponding opposite development was seen for S-PTH. S-ionized calcium was unaltered in all three treatment groups after two years.

The prevalence of vitamin D deficiency was lower than before the start of treatment in all three groups, with only five subjects with vitamin D deficiency two years after RYGB, one subject with vitamin D deficiency after SG, and seven subjects with vitamin D deficiency after medical treatment (S2 Table). These subjects had a mean S-25(OH)D of 35.1±14.9 nmol/L at baseline which decreased to 20.1±4.8 nmol/L two years after treatment.

Vitamin D insufficiency, S-25(OH)D < 50 nmol/l, was more prevalent in the medically treated group compared to the surgically treated groups (SMD>0.1 and p<0.01, Table 2). 16% of the medically treated subjects reported taking vitamin D supplementation after two years compared to 81% and 71% in RYGB and SG, respectively. Calcium supplementation was also more common after RYGB and SG (RYGB 91% and SG 79%) than after medical treatment (6%), SMD>0.1 and p<0.001 (Table 2). There were 38 subjects after RYGB and 36 subjects after SG who reported lower vitamin D supplement doses than the national recommendations (not shown). Self-reported supplement use at 6, 12, and 24 months after obesity treatment was lower at 24 months for both the operated and the medically treated groups (S1 Fig).

**Fig 3:**
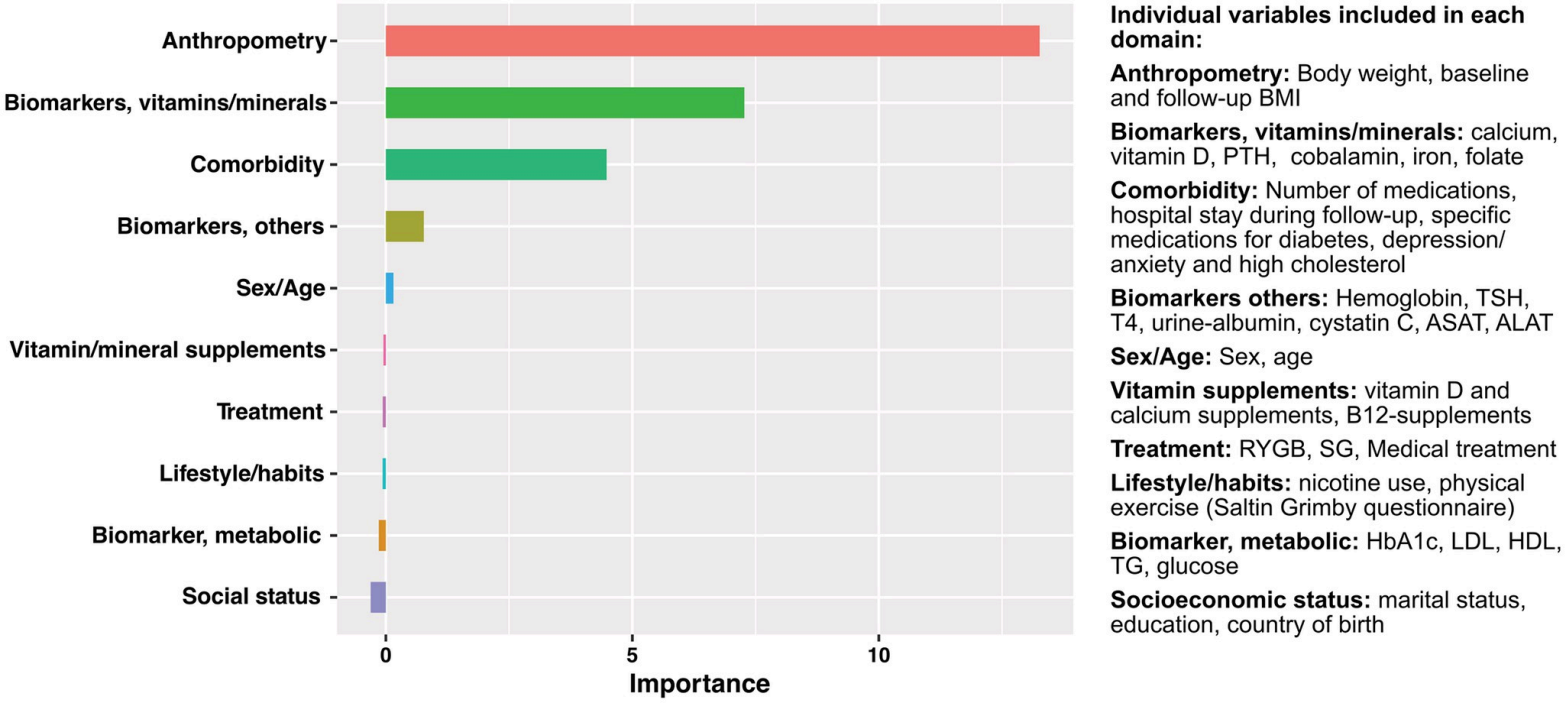
Predictive value of 10 clinical domains for the development of vitamin D deficiency after surgical and medical treatment for obesity. BMI, body mass index. PTH, parathyroid hormone. TSH, thyroid-stimulating hormone. T4, thyroxine. ALAT, alanine aminotransferase. ASAT, aspartate aminotransferase. RYGB, Roux-en-Y Gastric Bypass. SG, Sleeve Gastrectomy. HbA1c, glycated hemoglobin. HDL, high-density lipoprotein. LDL, low-density lipoprotein. TG, triglycerides.

In Fig 3 the relative importance of all 10 clinical domains regarding prediction for the development of vitamin D deficiency after obesity treatment, as well as an overview of the individual variables included in each domain, are presented. Follow-up BMI in the anthropometry domain was found to have the strongest predictive value for vitamin D deficiency after weight loss treatment in the conditional random forest model (Fig 3 and S2 Fig).

In the multivariate linear regression model, there was a significant positive association between S-25(OH)D after treatment and surgical treatment options (RYGB and SG), age, use of vitamin D supplements, and S-25(OH)D levels at baseline with p<0.05 (not shown). There was also a negative correlation between S-25(OH)D and S-PTH at two years (p<0.05). The association with follow-up BMI or country of birth was not significant (p>0.05).

## Discussion

The present study is, to our knowledge, the largest and only controlled study on vitamin D status after bariatric surgery in comparison to non-surgical treatment with a structured very-low-energy diet (VLED). The main finding was that there was no difference in risk for vitamin D deficiency and secondary hyperparathyroidism at the two-year follow-up between individuals undergoing surgical treatment and individuals on VLED.

All individuals with obesity in this study showed higher levels of S-25(OH)D and lower rates of vitamin D deficiency, vitamin D insufficiency, and secondary hyperparathyroidism two years after treatment. Furthermore, individuals undergoing RYGB and SG had significantly higher S-25(OH)D values than those treated with the VLED regimen. One explanation for the higher levels observed in individuals following RYGB and SG procedures may be attributed to the large difference in the use of vitamin supplements. The majority of surgically treated subjects reported usage of vitamin D and calcium supplementation while the corresponding use was merely 20% in the medically treated subjects at the two-year follow-up. The present study underscores the importance of vitamin D and calcium supplementation in preventing vitamin D deficiency after weight loss treatment. Post-surgical BMI and pre-operative vitamin D status have in previous studies been shown to correlate with vitamin D deficiency and secondary hyperparathyroidism following surgery [12, 30–35]. Similar to these previous findings, the present study found that post-surgical BMI had the strongest predictive value for vitamin D deficiency following treatment in the machine learning analyses. Machine learning was used to assess the predictive ability of several different variables on vitamin D deficiency at the 2-year follow-up. Using random forest allows for non-linear relationships and interactions of the different variables, which the regression models do not. This, and the lack of power, could explain the difference in results between the random forest analysis and the results of the multivariate regression analyses in this study.

Another finding was that vitamin D levels were lower in all individuals with obesity at the onset of the study, before treatment, compared to the population sample from the same region. There is today a well-acknowledged association between obesity and vitamin D insufficiency, which could explain our findings. Large epidemiologic studies, such as the Framingham Heart Study, have demonstrated an inverse correlation between high BMI and low S-25(OH)D [36]. However, the nature of this relationship is still not completely elucidated, and it does not appear to be strictly linear, as shown by Chan et al [34]. S-25(OH)D concentration appeared to flatten out once BMI approached 60 kg/m^2^. Several theories have been discussed and behavioral factors such as inadequate dietary habits and low sun exposure due to decreased physical activity, particularly outdoors, are among some. In a recent study by Ceolin et al., a significant association was demonstrated between obesity by adiposity (% fat) and a higher risk for both vitamin D deficiency and insufficiency [37]. This is in line with previous studies such as The Framingham Heart Study which indicated that low sun exposure alone was insufficient to explain the association, and instead, vitamin D sequestration in adipose tissue was considered the more likely mechanism underlying vitamin D deficiency in obesity [36]. Other studies found that volumetric dilution, rather than sequestration, best explained hypovitaminosis D in people with obesity [38, 39]. Furthermore, reduced CYP2R1 expression in the liver has also been proposed as another potential mechanism [40].

The present study found no significant difference in vitamin D status after RYGB and SG, respectively, two years post-operatively, despite the proposed malabsorptive component of the RYGB procedure. This was consistent with previous studies by Moizé et al. and Alexandrou et al. which found no difference in rates of vitamin D deficiency and secondary hyperparathyroidism between RYGB and SG two and four years following surgery, respectively [16, 41]. Similar to the present results, both mentioned studies reported no difference in supplement use between RYGB and SG [16, 41]. Therefore, individuals undergoing RYGB may not be at a higher risk of developing vitamin D deficiency than those undergoing SG during the first two years.

In general, most previous studies have shown increments in S-25(OH)D concentrations and lower rates of vitamin D deficiency but the results vary widely, with rates of vitamin D deficiency ranging from 6% to 94% postoperatively [10, 13, 15, 16, 30, 33, 35, 41, 42]. The same applies to secondary hyperparathyroidism with rates varying between 10% and 40% in the same studies. This discrepancy in results is most likely explained by using different definitions for vitamin D deficiency and regimens for vitamin D and calcium supplements, as well as differences in population characteristics, sample size, and follow-up time.

Current guidelines for vitamin D status after surgery recommend target levels for S-25(OH)D above 75 nmol/L [43], as a concentration of ≥ 75 nmol/L has been recognized as the optimal level for vitamin D homeostasis [44]. In our study, none of the treatment groups met this criterion at the two-year follow-up, with the medically treated group being farthest away from the target. This is in line with previous studies on vitamin D status after surgical treatment, in which most showed increments in S-25(OH)D concentrations but remained below the optimal level of 75 nmol/l [13]. This suggests that a more rigorous nutritional follow-up with more explicit and higher recommendations for vitamin supplementation is required.

Finally, only a few studies have compared persons with obesity to a random population sample regarding vitamin D status. Vitamin D deficiency was more prevalent in persons with obesity than in the reference population of the present study. However, supplement use was similar in the BASUN and WHO MONICA studies. These results are in line with a study by Goldner et al. in which individuals with obesity prior to surgery had higher rates of vitamin D deficiency compared to an aged-matched non-obese population from the same region [45]. The present study showed a significant difference in age with the reference population being older. However, age was not found to correlate with, or have a strong predictive value for vitamin D deficiency after treatment in our study. This is consistent with a meta-analysis by Pereira et al, in which age was found to have little influence on the inverse correlation between high BMI and low S-25(OH)D [46].

### Strength and limitations

No comparative study on medically and surgically treated persons with obesity regarding vitamin D status has to our knowledge previously been published. An important strength of the study was the use of a reference population that was randomly selected from the general population from the same region. A limitation could be that all the persons with obesity were recruited through medical referral which may affect the generalizability of the results on the entire population with obesity. However, participant and clinician involvement in the recruitment process of the present non-randomized study population represents real life and those seeking help. It is therefore a relevant representation of persons with obesity seeking treatment.

The reference group was neither age-matched nor concurrent with the study group and caution is therefore warranted when interpreting the between-group analyses. Because societal behaviors and systems are likely to be different at different points in time, the age disparity in addition to using a historical comparison group increases the risk of influence by confounding factors. Another limitation was the use of self-reported data regarding medications and supplements which may cause bias. At the two-year follow-up, vitamin D concentrations were missing for 27% of the subjects. This could cause a selection bias as subjects who completed the full follow-up might be more likely to adhere to or comply with medical instructions. There was, however, no great difference in clinical baseline data between the subjects who were lost to follow-up and those included in the two-year analyses.

The seasonal variation could have influenced the S-25(OH)D data in BASUN. However, the number of blood samples collected during sunny months was only around 10% at the follow-up which might not explain the total increase. The lack of data on dietary intake post-operatively could also be a potential confounder.

## Conclusion

Individuals undergoing surgical intervention for obesity, regardless of the method used, followed by vitamin D supplements were not at an increased risk for developing vitamin D deficiency compared to individuals receiving medical treatment at the two-year follow-up. In fact, vitamin D insufficiency was more prevalent in subjects with obesity after medical treatment with VLED. Future guidelines may consider including recommendations on vitamin D supplements for individuals on medical treatment for weight loss as well. Furthermore, persons with obesity seeking weight loss treatment are more likely to have insufficient vitamin D levels and secondary hyperparathyroidism than the general population.

## Supporting information

S1 Table Number (n) of subjects with missing data for each studied variable; anthropometric and social background data, physical activity, supplement use, and biochemical variables. (DOCX)

S2 Table Characteristics of subjects from the BASUN study with vitamin D deficiency (S-25(OH)D < 25 nmol/l) 2 years after treatment (n = 13). (DOCX)

S1 Fig Self-reported use of supplements from baseline up to the two-year follow-up. **A**. Vitamin D supplements. **B**. Calcium supplements.
(TIF)

S2 Fig Predictive value of each variable in the 10 clinical domains for the development of vitamin D deficiency after surgical and medical treatment for obesity. (TIF)

S1 File Raw data for BASUN. (XLSX)

S2 File Raw data for WHO-MONICA. (XLSX)
