# Supplementary material for: Effects of medical and surgical treatment on vitamin D levels in obesity
Source: PLoS One. 2023 Dec 22;18(12):e0292780. doi: 10.1371/journal.pone.0292780 (PMC10745143; doi:10.1371/journal.pone.0292780)
Supplement: S1 Table — (DOCX) [file pone.0292780.s001.docx]

| ***Supplementary table 1.*** Number (n) of subjects with missing data for each studied variable; anthropometric and social background data, physical activity, supplement use and biochemical variables. | | | |
| --- | --- | --- | --- |
|  | **BASUN baseline**  **(n = 971)** | **WHO MONICA,**  **Controls**  **(n = 414)** | **BASUN 2-year follow-up** |
| **Sex** | 3 | 0 | n/a |
| **Age** | 0 | 0 | n/a |
| **Body Mass Index, kg/m^2^** | 0 | 1 | 100 |
| **Married/Cohabiting** | 100 | 11 | n/a |
| **Completed** **Secondary** **school** | 117 | 34 | n/a |
| **Smoking** | 185 | 6 | n/a |
| **Physical** **exercise, Saltin Grimby** | 86 | 7 | n/a |
| **Number of medications per person** | 0 | 0 | n/a |
| **Vitamin D supplement** | 126 | 36 | 324 |
| **Calcium supplement** | 128 | n/a | 324 |
| **S-25(OH)D nmol/l** | 8 | 2 | 258 |
| **S-25(OH)D (nmol/l) <25** | 8 | 2 | 258 |
| **S-25(OH)D (nmol/l) <50** | 9 | 2 | 258 |
| **S-PTH (pmol/l)** | 23 | 0 | 259 |
| **S-PTH (pmol/l) > 6.9** | 23 | 0 | 259 |
| **S-ionized calcium (mmol/l)** | 66 | n/a | 276 |

*****Missing data for BASUN at baseline and WHO-MONICA. Missing data BASUN at 2-year follow-up compared to baseline. n/a.
