# Supplementary material for: Effects of medical and surgical treatment on vitamin D levels in obesity
Source: PLoS One. 2023 Dec 22;18(12):e0292780. doi: 10.1371/journal.pone.0292780 (PMC10745143; doi:10.1371/journal.pone.0292780)
Supplement: S2 Table — (DOCX) [file pone.0292780.s002.docx]

| *Supplementary table 2.* Characteristics of Patients from the BASUN study with Vitamin D Deficiency (S-25(OH)D < 25 nmol/l) 2 years after treatment (n=13). | | |
| --- | --- | --- |
|  | **Baseline** | **2-year follow-up** |
| Sex (female), n | 7 | |
| Age (years), mean (SD) | 44.5 (15.6) | 46.5 (15.6) |
| Body weight (kg), mean (SD) | 122.6 (22.9) | 94.9 (15.6) |
| BMI (kg/m²), mean (SD) | 41.2 (4.8) | 33.9 (5.1) |
| Bariatric treatment, n |  |  |
| Gastric bypass, n | 5 | |
| Sleeve gastrectomy, n | 1 | |
| Medical treatment, n | 7 | |
| Number of medications, mean (SD) | 4 (4.7) | 1.6 (3.1) |
| Vitamin D supplement, n | 1 | 3 |
| Calcium supplement, n | 0 | 3 |
| S-ionized calcium (mmol/l), mean (SD) | 1.2 (0.0) | 1.2 (0.0) |
| S-25(OH)D (nmol/l), mean (SD) | 35.1 (14.9) | 20.1 (4.8) |
| S-25(OH)D (nmol/l) <25, n | 3 | 13 |
| S-PTH (pmol/l), mean (SD) | 8.8 (9.2) | 5.9 (1.5) |
| S-PTH (pmol/l) > 6.9, n | 5 | 4 |
| Abbreviations: BMI, Body Mass Index; PTH, Parathyroid hormone; BASUN, BAriatric surgery SUbstitution and Nutrition study | | |
