# Supplementary figures and images for: Effects of medical and surgical treatment on vitamin D levels in obesity
Source: PLoS One. 2023 Dec 22;18(12):e0292780. doi: 10.1371/journal.pone.0292780 (PMC10745143; doi:10.1371/journal.pone.0292780)

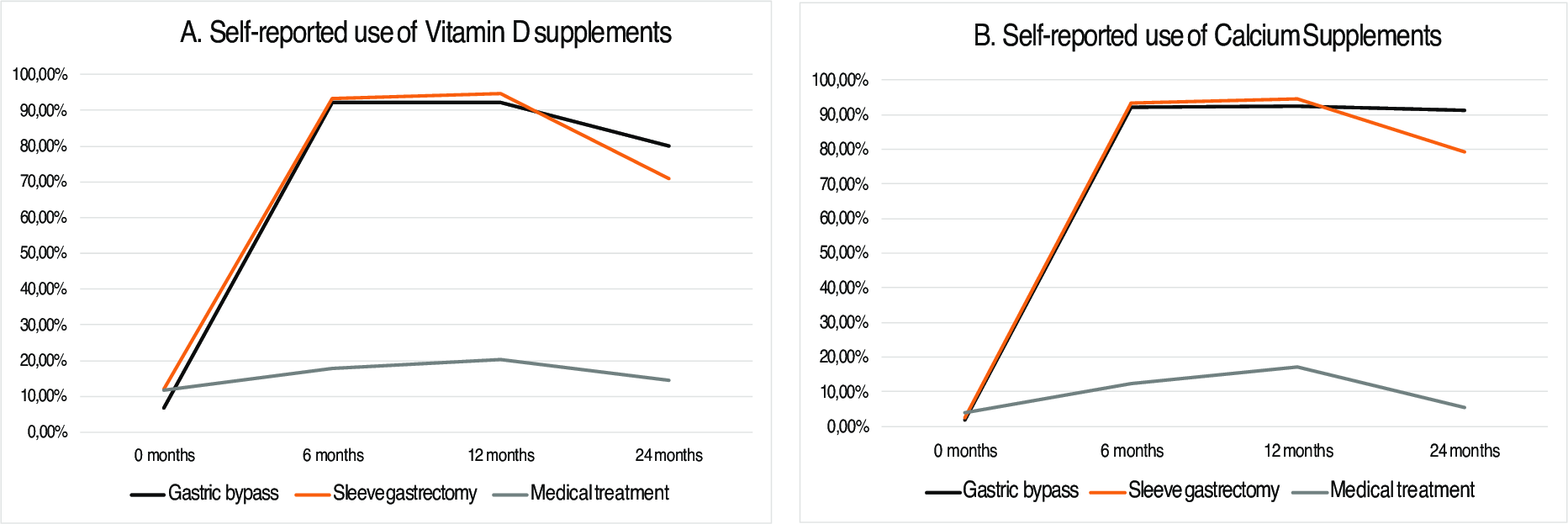

Supplement: S1 Fig — A. Vitamin D supplements. B. Calcium supplements. (TIF) [file pone.0292780.s003.tif]

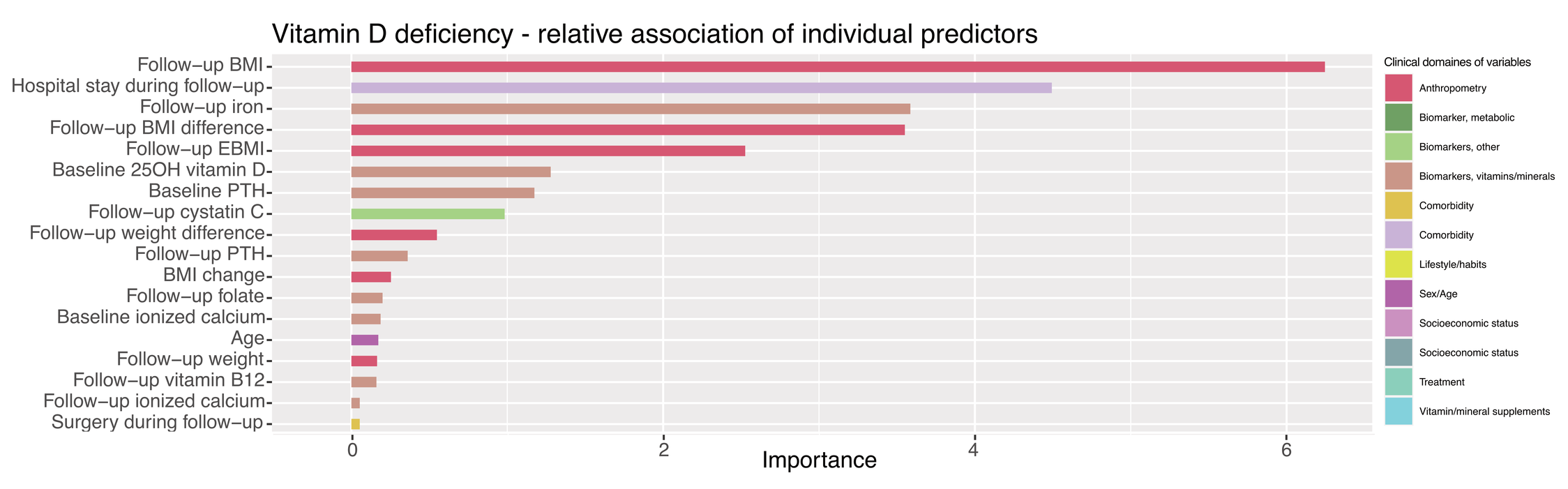

Supplement: S2 Fig — (TIF) [file pone.0292780.s004.tif]
